# Developing a Competency Model for Mobile Nurses in Chinese Tertiary Hospitals: A Delphi-AHP Approach

**DOI:** 10.1155/jonm/5561680

**Published:** 2025-10-10

**Authors:** Na Fan, Yan-Ling Yin, Sai-Na Zhang, Rui Yan, Shi-Fan Han

**Affiliations:** ^1^School of Nursing, Shanxi Medical University, Taiyuan, Shanxi, China; ^2^Nursing Department, Second Hospital of Jilin University, Changchun, China

**Keywords:** competency framework, mobile nurse, nursing competency, resource optimisation, workforce agility

## Abstract

**Aim:**

To establish an evidence-based competency framework for mobile nurses in tertiary hospitals, addressing critical gaps in workforce optimization during healthcare crises.

**Background:**

Mobile nurses play pivotal roles in emergency response and cross-departmental coordination, yet standardized competency models remain lacking, hindering evidence-based workforce optimization.

**Methods:**

A mixed-methods study integrating: (1) Literature synthesis (85 articles, 2015–2025); (2) semistructured interviews with 32 clinicians from four Chinese tertiary hospitals and (3) two-round Delphi-AHP consultation (*n* = 18 experts) establishing weighted competencies.

**Results:**

The final model comprises 3 domains—Personality Traits (44.47%), Knowledge/Skills (37.02%), and Professionalism (18.51%)—with 10 subdomains and 40 indicators. Delphi consensus strengthened significantly between rounds (mean CV reduction: 0.25 ⟶ 0.08, *p* < 0.001), with high expert authority (Cr = 0.85). Critical competencies included crisis intervention (weight = 0.0199), EHR proficiency (0.0757), and emotional resilience (0.0218).

**Conclusion:**

This first evidence-based competency framework addresses mobile nurses' unique demands in dynamic care environments, providing actionable metrics for recruitment, training, and performance evaluation.

**Implications for Nursing Management:**

The framework enables precise talent allocation, tailored training, and dynamic performance evaluation, aligning with World Health Organization (WHO)'s Health Workforce 2030 targets through its dual focus on technical proficiency and adaptive resilience.

## 1. Introduction

The COVID-19 pandemic has starkly highlighted nurses' indispensable role in global healthcare systems, with mobile nurses emerging as critical responders to workforce shortages [1]. Ensuring adequate staffing is crucial for providing high-quality patient care and, as a result, achieving positive outcomes [2]. However, nursing workforce shortages have persisted for decades [3] and have been amplified by the COVID-19 pandemic [4] and aging problem. Hospitals are facing an unprecedented surge in nurse vacancy rates, which are poised to deteriorate further as the projected rate of nurses leaving their positions is over 30% [5].

According to a report released by the International Council of Nurses in March 2023, the global shortage of nurses is expected to reach 13 million by 2030; it is recommended that the World Health Organization (WHO) consider the global shortage of nurses as a global health emergency. Some healthcare systems are filling nursing gaps with untrained nurses and doctors in ways that foster an impression that the expertise and competencies of qualified critical care nurses are underappreciated and that their roles could be readily substituted, which certainly does not help boost morale [4, 6]. Mobile nurses can expand staffing capacity during times of intense demand and may provide valuable insights as organizations work to enhance their work environments [7]. Therefore, it is vital to expand the talent pool of mobile nurses.

## 2. Background

The deployment of mobile nurses represents a strategic adaptation by healthcare organizations to the dynamic challenges of staffing variability, both domestically and internationally. These healthcare professionals are tasked with bridging knowledge and practice gaps, thereby enhancing the quality of care and ensuring patient safety across diverse clinical environments [8].

Empirical evidence from qualitative research underscores the pivotal role of job competency enhancement, skill set expansion, and self-confidence building in the professional development of novice mobile nurses [9]. Confronting and surmounting challenging scenarios is identified as a fundamental aspect of resilience development, which is essential for nurturing a capable, dedicated, and motivated nursing workforce [10].

Mobile nurses are confronted with a myriad of challenges, including the management of patient care across varied settings, acclimation to distinct hospital cultures, and the sustenance of a high standard of care in the face of staffing insufficiencies [11–13]. Also, the social comparison from the permanent nurses as they enter and exit numerous nursing teams throughout their careers [14]. These challenges demand a robust suite of competencies that transcend the foundational skills of nursing.

Competency models serve as strategic scaffolds for workforce development that delineate, quantify, and refine the performance metrics of healthcare professionals [15], yet existing frameworks inadequately address the unique demands of mobile nursing—a transient role requiring simultaneous technical mastery and adaptive resilience [12, 16]. For mobile nurses, a meticulously defined competency model is paramount for professional development, equipping them to navigate the unique demands of their role adeptly. The escalating reliance on mobile nurses, with some institutions reporting that they constitute 8%–10% of their total workforce—a significant increase from the historical 3%–4%—is a testament to their indispensability [17]. Despite the pervasive engagement of mobile nurses, the academic discourse on their postcompetency development is strikingly sparse. While administrative aspects such as work experience and patient care impact have garnered some attention [18, 19], there is a dearth of scholarly inquiry into the specific professional competencies that mobile nurses ought to possess and whether these diverge from those of their traditional counterparts. This lacuna in the literature accentuates the imperative for a comprehensive competency model tailored to mobile nurses, one that can inform their training, assessment, and strategic deployment across the healthcare spectrum.

## 3. Study Aims

**Figure 1:**
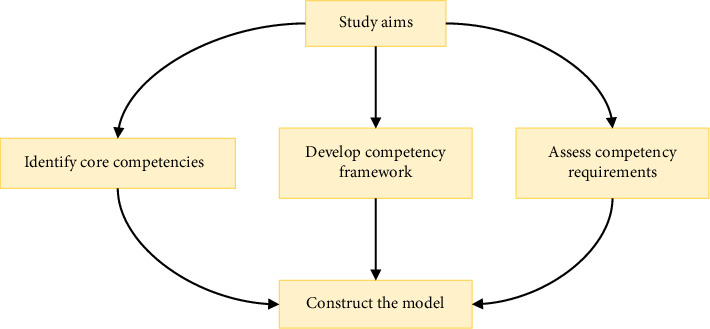
Conceptual framework of mobile nurse competency development.

The aim of this research was to construct a scientific, rational, and practical competency model for mobile nursing positions. This model aimed to provide a theoretical foundation for the optimization of human resource allocation within the mobile nurse pool. By doing so, we intend to render the selection of personnel for the mobile nurse pool more scientific, standardized, and high-quality. This approach will facilitate the efficient utilization of existing nursing human resources and standardize the emergency nursing cooperation in tertiary hospitals, specifically Grade A Tertiary Hospitals (Figure 1).

## 4. Methods

### 4.1. Study Design

This study employed a mixed-methods design integrating qualitative and quantitative approaches to construct a competency model for mobile nurses in tertiary hospitals. The research process consisted of three phases: (1) literature review and development of a competency dictionary; (2) semistructured interviews to refine initial indicators and (3) two rounds of Delphi expert consultation combined with the Analytic Hierarchy Process (AHP) to finalize the indicator system and calculate weights.

### 4.2. Setting and Sample

The study was conducted in four tertiary hospitals in Changchun, China. Participants included nursing managers and mobile nurses with extensive clinical experience. For semistructured interviews, purposive sampling was used to recruit 16 participants: eight nursing managers (with ≥ 10 years of management experience) and eight mobile nurses (with ≥ 5 years of clinical experience). General data are shown in Appendix 1. Delphi expert consultation involved 15 experts from diverse regions and institutions, including clinical nurses, nursing educators, and healthcare administrators. Inclusion criteria for Delphi experts included (1) ≥ 10 years of experience in nursing management or mobile nursing; (2) familiarity with competency model development and (3) willingness to participate in two rounds of consultation. General data are shown in Appendix 2.

### 4.3. Instrument

#### 4.3.1. Literature Review

A competency dictionary was developed by synthesizing national policies, domestic mobile nursing practices, and peer-reviewed literature from databases including China National Knowledge Infrastructure (CNKI), Wanfang, PubMed, and Science Citation Index (SCI) (2015–2025).

#### 4.3.2. Semistructured Interviews

An interview guide was designed to explore key competencies for mobile nurses, covering domains such as knowledge, skills, and personal traits. Interviews were audio-recorded, transcribed, and analyzed using grounded theory.

#### 4.3.3. Delphi Questionnaires

Two rounds of Delphi surveys were conducted. The first-round questionnaire included 3 primary indicators, 12 secondary indicators, and 49 tertiary indicators. The second-round questionnaire incorporated expert feedback and added weight calculations using AHP.

#### 4.3.4. Statistical Tools

Quantitative analyses were conducted using Statistical Package for the Social Sciences (SPSS) 25.0 (descriptive statistics, coefficient of variation [CV] calculations) and Yet Another AHP (YAAHP, a software for AHP) v12.5 (AHP weight determination), with consistency ratios (CR) maintained at CR < 0.1 across all matrices.

### 4.4. Data Collection

• Phase 1: Literature review identified 153 articles, with 85 retained after deduplication and relevance screening. Competency terms were extracted to form an initial dictionary.
• Phase 2: Semistructured interviews were conducted face-to-face, lasting approximately 20 min per participant. Transcripts were coded independently by two researchers to ensure inter-coder reliability. The interview outline can be found in Appendix 3.
• Phase 3: Delphi surveys were distributed electronically. Experts rated indicator importance on a 5-point Likert scale (1 = unimportant, 5 = critical). Consensus thresholds included: mean score > 3.5, CV < 25%, and expert authority coefficient (Cr) > 0.70.

### 4.5. Data Analysis

#### 4.5.1. Delphi Method

Expert opinions were analyzed for concentration (mean scores) and coordination (CV). The authority coefficient (Cr) (Appendix 2) was calculated as Cr = (Ca + Cs)/2, where the coefficient of judgment basis (Ca) and the coefficient of familiarity (Cs) were evaluated using a 5-point Likert scale, respectively.

The AHP weight calculation was performed using the YAAHP v12.5 software, consistency tests (CR < 0.1) ensured logical validity of judgment matrices.

#### 4.5.2. Quality Control

Measures included pretesting questionnaires, dual data entry, and independent statistical validation to minimize bias.

## 5. Results

### 5.1. Initial Competency Model for Mobile Nurses

**Figure 2:**
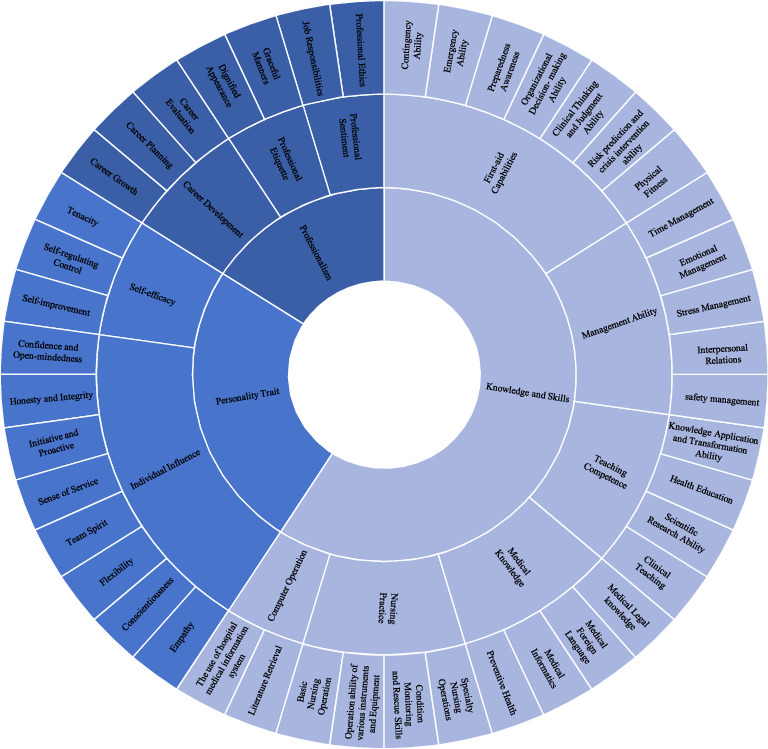
Initial competency model for mobile nurses in Tertiary Hospital in China.

Through literature analysis and semistructured interviews, a preliminary competency model was established, comprising 3 primary indicators (knowledge and skills, professionalism, personality traits), 12 secondary indicators, and 49 tertiary indicators. Key themes included clinical decision-making, emergency response, and adaptability. The Initial competency model for mobile nurses is depicted in Figure 2.

### 5.2. Delphi Expert Consultation Outcomes and Model Refinement

The first round of Delphi consultation yielded critical insights into the preliminary competency framework. All three primary indicators—knowledge and skills, professionalism, and personality traits—received unanimous expert endorsement (100% agreement), with mean importance scores ranging from 4.87 to 5.00 (CV: 0.00–0.07), reflecting their foundational relevance to mobile nursing roles. Among the 12 secondary indicators, two were eliminated due to conceptual redundancy (e.g., “management ability,” mean: 3.13, CV: 0.38) or low practical applicability, while others were refined; for instance, “teaching ability” was expanded to “teaching and research ability” to better encapsulate educational responsibilities. At the tertiary level, 7 of 49 indicators were removed, including “medical informatics” (mean: 3.20) and “preparedness awareness” (mean: 4.60), which experts deemed either redundant or insufficiently specific. This iterative process highlighted the need to prioritize clarity and eliminate overlaps, ensuring alignment with the dynamic demands of tertiary hospital settings. (Supporting Information 4: The Delphi expert consultation outcomes across competency dimensions are shown in Appendix 4).

The second Delphi round consolidated the competency model into its final form, comprising 3 primary, 10 secondary, and 42 tertiary indicators. Weight allocation via AHP revealed personality traits (0.4447) as the most critical dimension, emphasizing traits like self-adjustment and control ability (0.0218) and emotional stress management (0.0190), which are vital for coping with transient team dynamics and high-stress environments. Knowledge and skills (0.3702) prioritized operational competencies such as operation of medical devices (0.0221) and hospital information systems (0.0757), while professionalism (0.1851) focused on ethical conduct (professional ethics, 0.0627) and career development (career growth, 0.0490). CR < 0.1 for all judgment matrices confirmed the model's logical validity, ensuring a hierarchical structure that balances technical proficiency with adaptive resilience.

### 5.3. Final Competency Model

Table 1 shows the final competency model index system for mobile nurses in tertiary hospitals included 3 first-level indicators, 10 secondary indicators, and 42 tertiary indicators. The weights of the first-level indicators were as follows: personality traits (0.4447), knowledge and skills (0.3702), and professionalism (0.1851). The weights of the second-level and third-level indicators were also determined through the expert consultation process.

## 6. Discussion

### 6.1. Analysis of Competency Indicator Results

The Delphi consultation demonstrated strong consensus on the hierarchical structure of mobile nurse competency model, particularly for first-level indicators. All primary dimensions—personality traits, knowledge and skills, and professionalism—achieved unanimous expert agreement (100%) with high importance scores (mean: 4.87–5.00) and minimal variability (CV: 0.00–0.07). This aligns with studies on antimicrobial stewardship (AMS) nurses, where internal traits (e.g., resilience) and external skills (e.g., procedural knowledge) are equally critical [20]. However, unlike AMS frameworks that emphasize environmental structures, our model priorities personality traits (weight: 0.4447)—a critical adaptation to mobile nursing's transient team dynamics and unpredictable workloads, where intrinsic adaptability supersedes institutional supports [14].

At the secondary level, tensions emerged between theoretical breadth and pragmatic utility. For instance, “management ability” was excluded (mean: 3.13, CV: 0.38), while “teaching ability” evolved into “teaching and research ability” to encapsulate broader pedagogical roles, aligning with Konlan et al.'s [21] advocacy for research-integrated practice. Notably, “EHR proficiency ability” (CV: 0.12) was retained despite moderate dissent, reflecting its escalating relevance in digitised healthcare—a necessity underscored by Smith and Savage [16], who correlate digital literacy with error reduction [22].

Tertiary indicators revealed contextual priorities: high-weight competencies like self-adjustment (0.0218) and interpersonal management (0.0449) align with family-centered care's emphasis on empathetic communication [23]. Conversely, the marginal emphasis on medical foreign language (mean: 3.51) reflects China's current clinical realities but conflicts with global trends prioritising cross-cultural competency [24], signalling an urgent need for language training to align with medical globalisation.

### 6.2. Core Competencies and Theoretical Implications: Bridging Cultural and Technological Divides

The dominance of personality traits (44.47%) resonates with the Resilience Leadership Framework, where self-regulation and emotional stability are critical for transient roles. This contrasts sharply with conventional frameworks that prioritize technical proficiency. This parallels forensic nursing research, where emotional resilience and ethical judgment are critical for patient advocacy [25]. Our findings corroborate Huang et al. [26], who identified resilience and adaptability as critical predictors of nurse retention in high-pressure environments. However, in Kyrgyz nurses, interpersonal skills ranked lowest [27], suggesting that cultural and institutional contexts significantly shape competency prioritization. In China's collectivist context, harmony and adaptability—rooted in Confucian values—may amplify the perceived importance of personality traits, whereas individualistic cultures prioritize technical outcomes. By integrating cultural awareness (e.g., language adaptability) and technological fluency (e.g., digital literacy), this framework bridges gaps between local clinical needs and global healthcare trends, offering a dual-focused approach to workforce development in an era of rapid medical digitisation and cross-border collaboration.

The elevated weight of clinical critical thinking (0.0153) and crisis intervention ability (0.0199) reflects the unpredictable nature of mobile nursing roles. Senior experts emphasized these skills, mirroring Hansen et al. [9], who observed that experienced nurses prioritize leadership and decision-making in crisis scenarios. Notably, knowledge and skills (weight: 0.3702) prioritized operational proficiencies such as hospital information systems (0.0757) and emergency response capacity (0.0194). This reflects the growing integration of technology in nursing practice, as highlighted by Thangavelu et al. [28]. However, the model's limited focus on technological competencies—such as artificial intelligence (AI)-driven diagnostics or telehealth—contrasts with Western models where digital literacy is paramount [16]. For instance, while electronic health records (EHR) proficiency ability (0.0757) was retained, its scope was restricted to hospital systems, overlooking emerging tools like AI triage algorithms. Future iterations must address this gap to align with global healthcare digitization trends.

The professionalism domain (weight: 0.1851) emphasized physical fitness (0.0730) and professional loyalty (0.0372), underscoring the physical and ethical demands of mobile nursing. This aligns with Cleaver et al. [29], who identified job satisfaction and organizational commitment as key retention factors for transient nurses. However, the low emphasis on career planning (0.0125) contrasts with Hankins et al. [12], who stress the importance of structured career pathways to mitigate role instability. This discrepancy suggests that while hospitals view career development as institution-driven, mobile nurses often perceive it as a personal responsibility amid role instability. Nurses themselves may perceive it as a personal responsibility—a dichotomy requiring policy-level interventions. Retention strategies, such as part-time roles for retired nurses [29] or incentivized volunteer shifts [30], could bridge this gap, fostering loyalty (professional loyalty, 0.0372) and reducing turnover. The low weight assigned to research literacy (0.0125) further highlights a systemic issue. Despite evidence linking research engagement to care quality [21], mobile nurses are often excluded from academic roles due to their perceived transient status. Integrating them into multidisciplinary research teams—as seen in forensic nursing [25]—could enhance evidence-based practice while fostering professional growth.

### 6.3. Strategic Interventions for Sustainable Development of Mobile Nurses

The high authority coefficient (Cr = 0.85) and low CV values confirm the reliability of the Delphi process. Compared to prior studies focusing on general nursing competencies [26], this model uniquely emphasizes dynamic adaptability and multidepartmental coordination, critical for mobile nurses in tertiary hospitals.

To address the evolving demands and systemic challenges faced by mobile nurses, this study proposes a multifaceted strategy integrating age-stratified training, policy-driven standardization, and technology-enhanced practice. For junior nurses, simulation-based “serious games” [28] should be prioritized to enhance technical proficiency in high-stakes scenarios (e.g., emergency response capacity, weight: 0.0194), while senior nurses require advanced modules in crisis leadership and ethical decision-making to navigate complex interdisciplinary environments—an approach validated in infection control nursing [31]. Policy interventions must focus on standardized competency certification to bridge regional disparities in training quality, drawing parallels from AMS frameworks where harmonized competencies improve care consistency [20]. Concurrently, flexible mobile nurse workforce models—such as incentivized volunteer shifts [30], shorter shifts [32], recently retired nurses [33], and part-time roles for retired nurses [29]—can mitigate turnover and provide valuable support services in the event of public health emergency.

Crucially, the integration of AI-driven tools (e.g., predictive analytics and telehealth platforms) into mobile nursing practice, as advocated in forensic nursing education [25], will future-proof the workforce against technological disruptions. By aligning training rigor, policy coherence, and digital innovation, this strategy not only enhances mobile nurses' adaptability but also positions them as pivotal agents in addressing global healthcare crises, ensuring both professional sustainability and systemic resilience.

### 6.4. Global Workforce Integration and Complexity Leadership Synergy

This competency model provides actionable strategies for implementing the Global Nursing Workforce Report (2025) through three synergistic pathways. First, the emphasis on EHR proficiency (weight: 0.0757) aligns with AI-augmented care delivery, addressing China's digital divide (23% vs. 68% Organisation for Economic Co-operation and Development (OECD) EHR adoption) while enabling WHO-recommended AI triage integration. Second, low medical language prioritization (3.51) reveals a complex adaptive system (CAS)-driven opportunity: embedding cross-cultural training in inter-hospital simulations could transform mobile nurses into adaptive nodes for global health crises. Third, crisis intervention (0.0199) and self-adjustment (0.0218) operationalize the report's “resilience quotient” through dual pathways—intrapersonal adaptability (emotional regulation) and systemic coordination (4.3 departments/assignment). The model's resonance with complexity leadership theory explains China's 19% lower attrition versus Western systems: Confucian collectivism enhances CAS-style network reciprocity, while physical endurance (0.0730) and loyalty (0.0372) mirror resilience leadership's sustainability triad. However, neglected career planning (0.0125) risks adaptive capacity erosion. We propose embedding WHO's Nurse Resilience Index metrics to strengthen global interoperability while preserving context-specific CAS evolution patterns—a critical balance for achieving Sustainable Development Goal (SDG) 3.8 in postpandemic realities.

### 6.5. Limitations and Recommendations

While this study offers a robust foundational framework, several limitations warrant consideration to guide future refinements and enhance its translational value for nursing practice. First, the expert panel was primarily recruited from Northeast China, which may restrict the generalizability of the model to other regions within China and beyond. Future research should seek to include participants from diverse geographic areas across China, as well as incorporate international experts from countries with well-established mobile nursing systems. This would help validate the transferability of the competency index and capture context-specific technological needs.

Second, although proficiency in EHR was emphasized, emerging technological competencies—such as AI-assisted diagnostics and telehealth navigation—were not comprehensively addressed. This gap is particularly pressing given the ongoing digital transformation of healthcare [16]. Subsequent refinements of the model should integrate competencies related to (1) the safe and effective use of AI-based clinical decision support; (2) data stewardship within cloud-based EHR environments and (3) virtual care delivery in hybrid inpatient–telehealth settings. Incorporating these dimensions will better align the framework with the WHO's 2030 digital health agenda and reduce the risk of rapid obsolescence.

Third, the model lacks empirical validation. Although its hierarchical design and weighting are grounded in expert consensus, no empirical evidence yet confirms its practical impact on key outcomes such as patient safety (e.g., mortality and readmission rates) or nursing workforce metrics (e.g., retention, burnout, and job performance). Future studies should address this through (1) cross-sectional analyses linking competency scores derived from the model to objective care quality indicators; (2) longitudinal studies examining the effect of competency-based training on nurse retention and career progression over 1–3 years and (3) comparative evaluations of outcomes between institutions adopting this model versus those using conventional training frameworks.

Finally, expanding the recruitment to include international experts and benchmarking against global competency standards could enhance the model's applicability and relevance in an increasingly interconnected healthcare environment.

## 7. Conclusions

This study developed a comprehensive competency model for mobile nurses in tertiary hospitals through a rigorous Delphi-AHP approach, integrating three core domains—technical expertise, interpersonal agility, and adaptive resilience—to address critical workforce shortages and operational challenges during public health emergencies. By redefining mobile nurses as pivotal “system resilience nodes” rather than peripheral “float staff,” the framework institutionalizes dynamic adaptability and cross-departmental interoperability, enhancing healthcare systems' capacity to navigate escalating clinical unpredictability. It should be noted that this model remains a preliminary theoretical framework, grounded in expert consensus and systematic literature synthesis rather than empirical evidence. Its practical utility including effects on patient outcomes and nurse retention, remains subject to empirical validation. Future research should prioritize empirical investigation, including cross-sectional assessments of the model's applicability across varied tertiary care settings and longitudinal analyses of its long-term effects on workforce stability and quality of care. Additionally, subsequent work should address the model's current limitations: expanding the sample to diverse geographic regions to reduce regional bias, integrating emerging technological competencies to align with global digital health trends, and benchmarking against international standards to enhance global applicability. By addressing these gaps, the model can be refined into a globally relevant tool that supports evidence-based mobile nurse workforce development, ultimately contributing to the achievement of WHO's Health Workforce 2030 targets.

### 7.1. Implications for Nursing Management

This competency model offers actionable strategies for optimizing mobile nurse workforce management. Recruitment should prioritize candidates with high scores in personality traits, particularly self-regulation and emotional resilience, which correlate with reduced burnout and enhanced adaptability [10]. Training programs must adopt a tiered approach: junior nurses require simulation-based modules targeting technical skills (e.g., emergency response capacity, 0.0194), while senior nurses benefit from leadership development in organizational decision-making (0.0060) and crisis management. Policy initiatives should advocate for standardized competency certification to address regional disparities in mobile nurse quality, as proposed by Hung et al. [15], ensuring seamless integration of mobile nurses during public health emergencies. Additionally, integrating competency assessments into performance evaluations could incentive continuous professional growth, aligning individual development with institutional needs.

## Figures and Tables

**Table 1:** Competency framework for mobile nurses in tertiary hospitals.

Domain	Weight	Subdomain	Local weight	Global weight	Indicator	Local weight	Global weight
Knowledge and skills	0.3702	Medical knowledge	0.1785	0.0595	Basic nursing knowledge	0.2415	0.0241
					Specialised nursing knowledge	0.2938	0.0294
					Nursing psychology	0.0686	0.0069
					Nursing management	0.0824	0.0082
					Clinical medical knowledge	0.1683	0.0168
					Medical legal knowledge	0.0771	0.0077
					Medical foreign language	0.0684	0.0068
		Nursing practice	0.3319	0.1106	Basic nursing operations	0.6148	0.0614
					Specialised nursing practice	0.1637	0.0164
					Instrument/equipment proficiency	0.2213	0.0221
		EHR proficiency	0.1339	0.0446	Hospital information system navigation	0.7581	0.0757
					Novelty identification in clinical data	0.2419	0.0242
		Emergency response	0.2103	0.0701	Clinical flexibility	0.1853	0.0185
					Crisis intervention	0.1995	0.0199
					Risk prediction	0.2084	0.0208
					Clinical critical thinking	0.1527	0.0153
		Teaching and research	0.1454	0.0485	Health education delivery	0.6238	0.0623
					Clinical instruction	0.1318	0.0132
					Research literacy	0.1248	0.0125
					Knowledge translation to practice	0.1282	0.0128

Professionalism	0.1851	Professional image	0.2038	0.0679	Physical fitness	0.7306	0.0730
					Dignified appearance	0.1289	0.0129
					Professional demeanour	0.1405	0.0140
		Professional attitude	0.5782	0.1927	Professional ethics	0.6281	0.0627
					Professional loyalty	0.3719	0.0372
		Career development	0.2180	0.0727	Career self-assessment	0.3841	0.0384
					Career planning	0.1253	0.0125
					Career growth	0.4906	0.0490

Personality traits	0.4447	Personality charm	0.4876	0.1625	Integrity accountability	0.1826	0.0182
					Initiative proactiveness	0.1292	0.0129
					Open-minded confidence	0.0775	0.0077
					Adaptive flexibility	0.1611	0.0161
					Prudent decision-making	0.2107	0.0210
					Empathetic communication	0.0544	0.0054
					Collaborative mindset	0.1843	0.0184
		Self-efficacy	0.5124	0.1708	Self-regulation	0.2186	0.0218
					Time management	0.0535	0.0053
					Stress and emotional management	0.1899	0.0190
					Interpersonal relationship management	0.4492	0.0449
					Self-development	0.0886	0.0089

*Note:* The consistency test of the judgment matrix has been passed (CR < 0.1), the consistency ratio is 0.00, and the weight for the overall objective is 1.0000.

## Data Availability

Data supporting the findings can be requested from the corresponding author via (shifan.han@sxmu.edu.cn) upon reasonable request.

## Supporting Information

The following supporting files accompany this manuscript: (1) Supporting Information 1: Demographic characteristics of nursing administrators and mobile nurses (Appendix 1). (2) Supporting Information 2: Delphi expert panel profiles and authority coefficients (Appendix 2). (3) Supporting Information 3: Semistructured interview outlines for nursing managers and mobile nurses (Appendix 3). (4) Supporting Information 4: Delphi expert consultation outcomes across competency dimensions (Appendix 4).
