# Supplementary material for: Developing a Competency Model for Mobile Nurses in Chinese Tertiary Hospitals: A Delphi-AHP Approach
Source: J Nurs Manag. 2025 Oct 10;2025:5561680. doi: 10.1155/jonm/5561680 (PMC12534139; doi:10.1155/jonm/5561680)
Supplement: Supporting Information — Additional supporting information can be found online in the Supporting Information section. [file 5561680.f1.docx]

**Appendix**

**Appendix 1：**

**Table 1.** Demographic Characteristics of Nursing Administrators (n=8)

| **Participant ID** | **Expertise Area** | **Age (years)** | **Professional Title** | **Education** | **Position** | **Experience (years)** |
| --- | --- | --- | --- | --- | --- | --- |
| N1 | Nursing Management | 51 | Chief Nurse | MSc | Vice President | 30 |
| N2 | Nursing Management | 50 | Chief Nurse | MSc | Deputy Director, Nursing Dept | 28 |
| N3 | Clinical Nursing | 53 | Associate Chief Nurse | MSc | Head Nurse | 30 |
| N4 | Clinical Nursing | 51 | Associate Chief Nurse | MSc | Head Nurse | 27 |
| N5 | Clinical Nursing | 48 | Associate Chief Nurse | MSc | Head Nurse | 29 |
| N6 | Clinical Nursing | 39 | Senior Nurse | MSc | Head Nurse | 20 |
| N7 | Clinical Nursing | 47 | Associate Chief Nurse | MSc | Head Nurse | 25 |
| N8 | Clinical Nursing | 52 | Associate Chief Nurse | MSc | Head Nurse | 29 |
| **Mean ± SD** |  | 48.88 ± 4.45 |  |  |  | 27.25 ± 3.37 |

**Table 2.** Demographic Characteristics of Mobile Nurses (n=8)

| **Participant ID** | **Gender** | **Age (years)** | **Professional Title** | **Education** | **Marital Status** | **Experience (years)** |
| --- | --- | --- | --- | --- | --- | --- |
| N1 | Female | 26 | Nurse | MSc | Unmarried | 7 |
| N2 | Female | 29 | Nurse | BSc | Unmarried | 9 |
| N3 | Female | 29 | Nurse | BSc | Married | 11 |
| N4 | Male | 31 | Senior Nurse | MSc | Unmarried | 8 |
| N5 | Male | 30 | Nurse | BSc | Married | 9 |
| N6 | Female | 28 | Senior Nurse | MSc | Unmarried | 7 |
| N7 | Female | 30 | Nurse | MSc | Unmarried | 6 |
| N8 | Female | 31 | Senior Nurse | MSc | Married | 8 |
| **Mean ± SD** |  | 29.25 ± 1.67 |  |  |  | 8.13 |

**Appendix 2：**

**Table 3.**Demographic and Professional Characteristics of Expert Panel Participants (*N* = 15)

| **Characteristics** | ***n*** | **Percentage (%)** |
| --- | --- | --- |
| **Age (years)** |  |  |
| 30–39 | 2 | 13.3 |
| 40–49 | 9 | 60.0 |
| ≥50 | 4 | 26.7 |
| **Position** |  |  |
| Vice President | 1 | 6.7 |
| Director of Teaching Office | 1 | 6.7 |
| Director of Nursing Dept. | 3 | 20.0 |
| Department Head Nurse | 2 | 13.3 |
| Nurse Manager | 5 | 33.3 |
| Nursing Educator | 2 | 13.3 |
| Public Health Educator | 1 | 6.7 |
| **Professional Title** |  |  |
| Senior | 6 | 40.0 |
| Associate Senior | 7 | 46.7 |
| Intermediate | 2 | 13.3 |
| **Education** |  |  |
| Bachelor’s | 2 | 13.3 |
| Master’s | 10 | 66.7 |
| Doctoral | 3 | 20.0 |
| **Work Scope** |  |  |
| Clinical Medicine | 1 | 6.7 |
| Clinical Nursing | 6 | 40.0 |
| Nursing Education | 2 | 13.3 |
| Nursing Management | 5 | 33.3 |
| Other | 1 | 6.7 |
| **Work Experience (years)** |  |  |
| 10–19 | 4 | 26.7 |
| 20–29 | 7 | 46.7 |
| ≥30 | 4 | 26.7 |

Table 4. Expert Authority Coefficient Scores (Cr)**

| Expert ID | Judgment Score* | Familiarity Score† | Authority Coefficient (Cr)‡ |
| --- | --- | --- | --- |
| 1 | 0.870 | 0.800 | 0.835 |
| 2 | 0.850 | 1.000 | 0.925 |
| 3 | 0.800 | 0.800 | 0.800 |
| 4 | 0.870 | 0.800 | 0.835 |
| 5 | 0.870 | 0.600 | 0.735 |
| 6 | 0.770 | 0.800 | 0.785 |
| 7 | 0.850 | 1.000 | 0.925 |
| 8 | 0.850 | 1.000 | 0.925 |
| 9 | 0.870 | 1.000 | 0.935 |
| 10 | 0.950 | 1.000 | 0.975 |
| 11 | 0.770 | 0.800 | 0.785 |
| 12 | 0.720 | 0.800 | 0.760 |
| 13 | 0.970 | 1.000 | 0.985 |
| 14 | 0.770 | 0.800 | 0.785 |
| 15 | 0.720 | 0.800 | 0.760 |

**note**:Group Cr = ΣCr/15|0.833|0.867|0.850
Judgment Score: Rated on a 0.0–1.0 scale (1.0 = highest theoretical/practical relevance).
†Familiarity Score: 0.0 = unfamiliar; 0.5 = moderately familiar; 1.0 = fully familiar.
‡Authority Coefficient: Calculated as (Judgment Score + Familiarity Score)/2.

**Appendix 3：Semi - structured Interview Outline**

#### I. Questionnaire and Interview Outline for Nursing Managers' Basic Information

Dear teacher,

Hello!

It is a great honor that you can participate in this research, which enables the smooth progress of the project. This interview mainly focuses on "The Construction of the Competency Indicator System for Mobile Nurses". It will take about 20 minutes of your time. We will keep your information strictly confidential. Thank you for your participation!

**1.1Basic Information**

**Questionnaire on Nursing Managers' Basic Information**

Gender:

Age:

Professional Title:

Educational Background:

Research Field:

Years of Work:

Position:

Email:

**1.2Interview Outline**

From your personal perspective, please talk about your understanding of the characteristics of the nursing work of mobile nurses.

What abilities or qualities do you think mobile nurses should possess?

What are the post - competency indicators that you think distinguish mobile nurses from ordinary nurses?

What suggestions and opinions do you have on the construction of the competency indicator system for mobile nurses?

#### 2. Questionnaire and Interview Outline for Mobile Nurses' Basic Information

Dear nursing expert,

Hello!

It is a great honor that you can participate in this research, which enables the smooth progress of the project. This interview mainly focuses on "The Construction of the Competency Indicator System for Mobile Nurses". It will take about 20 minutes of your time. We will keep your information strictly confidential. Thank you for your participation!

**2.1Basic Information**

**Questionnaire on Mobile Nurses' Basic Information**

Gender:

Age:

Marital Status:

Educational Background:

Professional Title:

Hospital Grade:

Years of Work:

Years of Working as a Mobile Nurse:

Working Department:

Email:

Award - winning Situation:

**2.2Interview Outline**

Based on your work experience, what abilities or qualities does an excellent mobile nurse need to possess?

In which aspects do you think the most important post - competency indicators of mobile nurses are reflected?

In your opinion, what post - competency indicators do mobile nurses need to cultivate in the future?

**Appendix 4：**

**Table 5.** First-Round Delphi Consensus Results for Primary Competency Dimensions of Mobile Nurses

| **Primary Dimension** | **Experts (n)** | **Consensus Rate (%)** | **Importance (Mean ± SD)** | **Coefficient of Variation (CV)** |
| --- | --- | --- | --- | --- |
| Knowledge & Skills | 15 | 100 | 5.00 ± 0.00 | 0.00 |
| Professionalism | 15 | 100 | 4.87 ± 0.35 | 0.07 |
| Personality Traits | 15 | 100 | 4.93 ± 0.26 | 0.05 |

Table 6. First-Round Delphi Consensus Results for Secondary Competency Dimensions of Mobile Nurses

| Secondary Dimension | Consensus Rate (%) | Importance (Mean ± SD) | Coefficient of Variation (CV) | Revision Outcome |
| --- | --- | --- | --- | --- |
| Nursing Science Knowledge | 100 (15/15) | 5.00 ± 0.00 | 0.00 | Merged into "Medical Knowledge" |
| Medical Knowledge | 87 (13/15) | 4.80 ± 0.41 | 0.09 | Retained |
| Nursing Practice Competency | 100 (15/15) | 5.00 ± 0.00 | 0.00 | Retained |
| Information Application* | 87 (13/15) | 3.67 ± 0.82 | 0.22 | Renamed from "Computer Skills" |
| Management Ability | 87 (13/15) | 3.13 ± 1.19 | 0.38 | Deleted |
| Emergency Response | 100 (15/15) | 4.87 ± 0.35 | 0.07 | Retained |
| Teaching & Research Ability* | 67 (10/15) | 3.60 ± 0.82 | 0.22 | Expanded from "Teaching Ability" |
| Professional Image* | 93 (14/15) | 4.20 ± 0.68 | 0.16 | Renamed from "Professional Etiquette" |
| Professional Mindset* | 93 (14/15) | 4.33 ± 0.62 | 0.14 | Renamed from "Professional Sentiment" |
| Career Development | 73 (11/15) | 3.87 ± 0.74 | 0.19 | Retained |
| Personality Charm* | 87 (13/15) | 4.47 ± 0.52 | 0.12 | Renamed from "Personal Influence" |
| Self-Efficacy | 80 (12/15) | 4.13 ± 0.35 | 0.09 | Retained |

Notes:Consensus rate calculated as agreeing experts/total experts.*Dimensions with wording revisions based on expert feedback.CV = (SD/Mean) × 100%.

Table 7. First-Round Delphi Consensus Results for Tertiary Competency Dimensions of Mobile Nurses

| Tertiary Dimension | Consensus Rate (%) | Importance (Mean ± SD) | Coefficient of Variation (CV) | Revision Outcome |
| --- | --- | --- | --- | --- |
| Knowledge & Skills |  |  |  |  |
| Basic Nursing Science | 100 (15/15) | 4.80 ± 0.41 | 0.09 | Retained |
| Specialised Nursing Science | 100 (15/15) | 4.73 ± 0.46 | 0.10 | Retained |
| Nursing Psychology | 73 (11/15) | 4.07 ± 0.26 | 0.06 | Retained |
| Nursing Management* | 60 (9/15) | 3.93 ± 0.46 | 0.12 | Renamed from "Course of Nursing Management" |
| Preventive Health Science | 80 (12/15) | 4.27 ± 0.46 | 0.11 | Retained |
| Medical Informatics | 60 (9/15) | 3.20 ± 0.86 | 0.27 | Deleted |
| Medical Legal Knowledge | 87 (13/15) | 4.73 ± 0.46 | 0.10 | Retained |
| Medical Foreign Language | 67 (10/15) | 3.73 ± 0.46 | 0.12 | Retained |
| Nursing Practice |  |  |  |  |
| Basic Nursing Operations | 100 (15/15) | 4.87 ± 0.35 | 0.07 | Retained |
| Specialised Nursing Operations | 100 (15/15) | 4.73 ± 0.46 | 0.10 | Retained |
| Equipment Proficiency | 93 (14/15) | 4.87 ± 0.35 | 0.07 | Retained |
| HIS Navigation* | 87 (13/15) | 3.93 ± 0.46 | 0.12 | Renamed from "Hospital Medical Information System" |
| Novelty Identification* | 60 (9/15) | 2.93 ± 0.46 | 0.16 | Renamed from "Literature Retrieval" |
| Emergency Response |  |  |  |  |
| Clinical Flexibility | 93 (14/15) | 4.73 ± 0.46 | 0.10 | Retained |
| Crisis Intervention | 93 (14/15) | 4.87 ± 0.35 | 0.07 | Retained |
| Risk Prediction | 87 (13/15) | 4.73 ± 0.46 | 0.10 | Retained |
| Clinical Critical Thinking* | 80 (12/15) | 4.53 ± 0.52 | 0.11 | Renamed from " Clinical Thinking and Judgment Ability " |
| Organizational Decision-Making | 67 (10/15) | 3.93 ± 0.59 | 0.15 | Retained |
| Professionalism |  |  |  |  |
| Physical Fitness | 67 (10/15) | 4.87 ± 0.35 | 0.07 | Moved to "Professional Image" |
| Health Education | 73 (11/15) | 4.07 ± 0.26 | 0.06 | Retained |
| Professional Demeanour | 93 (14/15) | 4.87 ± 0.35 | 0.07 | Retained |
| Professional Ethics | 93 (14/15) | 4.73 ± 0.46 | 0.10 | Retained |
| Professional Loyalty* | 87 (13/15) | 4.67 ± 0.49 | 0.10 | Renamed from "Job Responsibilities" |
| Career Self-Assessment | 73 (11/15) | 3.93 ± 0.59 | 0.15 | Retained |
| Career Planning | 60 (9/15) | 3.07 ± 0.70 | 0.23 | Retained |
| Career Growth | 80 (12/15) | 3.87 ± 0.35 | 0.09 | Retained |
| Personality Traits |  |  |  |  |
| Integrity Accountability | 100 (15/15) | 4.87 ± 0.35 | 0.07 | Retained |
| Initiative Proactiveness | 100 (15/15) | 4.87 ± 0.35 | 0.07 | Retained |
| Open-Minded Confidence | 100 (15/15) | 4.67 ± 0.64 | 0.14 | Retained |
| Adaptive Flexibility | 93 (14/15) | 4.60 ± 0.51 | 0.11 | Retained |
| Service Orientation | 100 (15/15) | 4.80 ± 0.41 | 0.09 | Retained |
| Prudence in Practice | 100 (15/15) | 4.73 ± 0.59 | 0.13 | Retained |
| Empathetic Communication | 100 (15/15) | 4.73 ± 0.46 | 0.10 | Retained |
| Team Collaboration | 100 (15/15) | 5.00 ± 0.00 | 0.00 | Retained |
| Self-Regulation | 93 (14/15) | 4.27 ± 0.46 | 0.11 | Retained |
| Resilience* | 87 (13/15) | 4.47 ± 0.52 | 0.12 | Deleted |
| Self-Development | 80 (12/15) | 4.60 ± 0.51 | 0.11 | Retained |

notes :
HIS = Hospital Information System; CV = Coefficient of Variation.
Dimensions with CV ≥0.20 underwent wording revisions per expert feedback.
